# Inequality in uptake of isoniazid prevention therapy and Mantoux test among pregnant women with HIV in the Eastern Cape, South Africa

**DOI:** 10.1186/s12889-019-7769-y

**Published:** 2019-10-29

**Authors:** Oladele Vincent Adeniyi, Nonkosi Selanto-Chairman, Eyitayo Omolara Owolabi, Anthony Idowu Ajayi, Dominique Kabengele Kayembe, Daniel Ter Goon, Avramovic Gordana, John Lambert

**Affiliations:** 1 Department of Family Medicine & Rural Health, Faculty of Health Sciences, Walter Sisulu University, Mthatha/East London Hospital Complex, Cecilia Makiwane Hospital, East London, South Africa; 2 Department of Public Health, Faculty of Health Sciences, University of Fort Hare, East London, South Africa; 3 Department of Nursing Sciences, Faculty of Health Sciences, University of Fort Hare, East London, South Africa; 4 Department of Sociology, Faculty of Social Sciences & Humanities, University of Fort Hare, East London, South Africa; 5 Department of Family Medicine & Rural Health, Faculty of Health Sciences, Walter Sisulu University, Mthatha/East London Hospital Complex, Cecilia Makiwane Hospital, East London, South Africa; 6 Department of Nursing Sciences, Faculty of Health Sciences, University of Fort Hare, East London, South Africa; 7 Department of Infectious Diseases, Medicine and Sexual Health. Mater, Rotunda and University College, Dublin, Ireland; 8 Department of Infectious Diseases, Medicine and Sexual Health. Mater, Rotunda and University College, Dublin, Ireland; 9 Population Dynamics and Sexual and Reproductive Health Unit, African Population and Health Research Center, Nairobi, Kenya

**Keywords:** HIV-associated tuberculosis, Isoniazid prevention therapy, Mantoux test, Maternal mortality, Tuberculosis-associated morbidity, Tuberculosis-associated mortality

## Abstract

**Background:**

HIV-associated tuberculosis (TB) is a major cause of death among pregnant women in South Africa. Isoniazid prevention therapy (IPT) strategy was implemented in South Africa concurrently with life-long antiretroviral therapy (ART) to reduce the TB-associated morbidity and mortality in individuals living with HIV. This study assessed the extent of the implementation of IPT and the performance of the Mantoux test by geographic settings of health facilities and residences of pregnant women living with HIV in the Eastern Cape, South Africa.

**Methods:**

We conducted a data analysis of 1709 pregnant women enrolled in the new electronic database of the prevention of mother-to-child transmission programme of the East London Prospective Cohort Study. Relevant data on place of residence and antenatal care, performance of the Mantoux test and subsequent initiation of IPT were obtained. Descriptive and inferential statistics were employed to analyse the geographical variations and accessibility to Mantoux test and IPT.

**Results:**

The analysis shows that Mantoux test was performed on 803 pregnant women (47%) with significant geographical variation. After controlling for relevant covariates, pregnant women who resided in rural areas (AOR:0.63; CI: 0.47–0.84) compared to those who resided in urban areas were significantly less likely to receive Mantoux test. The rate of uptake of IPT was 79% with significant geographic variations. In the unadjusted model, rural place of residence (UOR:0.68; CI: 0.49–0.96) was independently associated with lower likelihood of uptake of INH prophylaxis; however, the effect was not significant after controlling for important covariates.

**Conclusions:**

The high uptake rate of isoniazid prevention therapy in pregnant women living with HIV at the study sites is commendable; however, concerted efforts are needed to address the inequality gaps in the roll-out of IPT. Poor performance of Mantoux test is a serious concern and requires the attention of TB programme managers and other relevant authorities.

## Background

South Africa has the highest global burden of HIV and tuberculosis (TB) epidemic globally, with more than 300,000 TB cases notified annually [1]. Since high HIV infection drives the growing burden of TB (epidemiological and biological link), South Africa is confronted with a huge burden of HIV and TB co-infection, especially among women [2]. With over seven million people living with HIV, and a prevalence of 30.2% among pregnant women in South Africa, with TB/HIV co-infection rate of 57%, there is a definite cause for public health concern. The prevalence of TB in HIV-infected pregnant women is similar to the general population, approximately 795/100,000 [2]. TB is the leading cause of maternal mortality in South Africa; accounting for 26.2% in non-pregnancy related infections [3]. The Eastern Cape Province has the second highest rate of TB cases, after Kwazulu-Natal, and is one of the three provinces lagging behind in the battle against TB and HIV in South Africa; others being the Northern Cape and North West Provinces [4].

Emerging evidence has shown that women of childbearing age are particularly susceptible to TB during pregnancy and this contributes significantly to the burden of maternal morbidity and mortality [2, 5, 6]. Besides, TB increases the risk of HIV transmission from the mother to the unborn child, abortion, premature delivery, post-partum haemorrhage, and pre-eclampsia [7, 8]. Various immunological changes increase the risk of progressing from latent TB infection to active TB disease among pregnant women [9]. This risk is particularly higher among HIV-infected pregnant women, a rate ten times higher than that found among non-HIV-infected pregnant women [5] and this has made the prevention of TB among HIV-infected pregnant women a global priority. Also, there is a significant risk of adverse infant outcomes such as low birth weight, prematurity and overall infant mortality [6, 10–12].

Combined antiretroviral therapy (ART) has been shown to reduce the risk of TB in HIV-infected individuals, including pregnant women; however, the incidence rate is still higher than acceptable levels. As such, the use of ART only is considered insufficient [13–16]. In the light of this, the Isoniazid Prevention Therapy (IPT) was introduced to augment ART. IPT is an essential public health strategy for prevention of TB among HIV infected individuals [17]. The IPT, with or without ART, has been shown in various randomised controlled trials and Cochrane reviews to significantly reduce the risk of developing active TB among those living with HIV who have latent TB infection [18–22]; with a 70 to 90% reduction. Studies in South Africa have also documented the effectiveness and safety of IPT, even among pregnant women and children [23, 24]. However, recent report by Gupta et al. [25] highlighted concerns of hepatotoxicity and adverse fetal outcomes following initiation of INH in pregnant and post-partum women on concomitant ART.

Globally, an increase in the uptake of IPT is recorded, however, the rate of uptake is still below acceptable levels, especially in the resource-poor settings [26, 27]. The World Health Global TB Report for 2015 showed that only 2.5% individuals living with HIV received IPT [28]. Studies conducted in resource-limited settings in some parts of sub-Saharan Africa, specifically Addis-Ababa, Ethiopia have also reported low rates of IPT uptake ranging from 32 to 34% [29, 30]. A report on the uptake of IPT in South Africa also highlighted a low level of IPT uptake [31]. In order for South Africa to reduce the incidence of TB from 9800 per million in 2010 to 1500 per million in 2050 and TB-associated mortality from 2180 per million in 2010 to 200 per million in 2050, IPT must be scaled up to 75% by 2035 [32]. Many other studies across Africa reported rates ranging from 54% in a health district of Zimbabwe [33] to 77% in an urban health centres of Kenya [34]. The recorded low uptakes, irrespective of the geographical settings, have been linked to patients’ related factors such as non-adherence, non-disclosure of HIV serostatus and lack of social support [35, 36] and health care related factors such as the levels of knowledge of the health care providers, and facilities being out of stock, amongst others [33, 37].

In 2014, the South African National Department of Health implemented the World Health Organization (WHO) recommendation on IPT [38]; 36 months for HIV-infected adults/adolescents with positive Mantoux test results and 12 months for Mantoux test negative individuals including pregnant women. This strategy was integrated into the mainstream HIV care services which included the intensified TB symptom screening. All patients, including pregnant women, receive TB symptom screening at diagnosis of HIV, following which the Mantoux test is performed once active TB has been excluded. Patients return after 48–72 h for reading of the Mantoux test. Isoniazid 300 mg is prescribed by the attending clinician on the treatment sheet. The duration of IPT is documented in the medical records of each patient.

Since the introduction of the IPT guideline [38], there has been no published data on the current rate of implementation of IPT and the performance of the Mantoux test (a form of tuberculin skin test) in the Eastern Cape, and especially, among pregnant women in South Africa. We aimed to assess the current uptake of IPT by geographic settings of health facilities and residences of pregnant women living with HIV in the Eastern Cape, South Africa. In addition, this study evaluated the performance of the Mantoux test as a measure of compliance with the IPT guideline. Findings of this study might help to identify the gaps in the implementation of IPT by programme managers and primary health care facilities and might inform the crafting of strategies to scale up the uptake of IPT in the region.

## Methods

### Study design

This is a sub-analysis of a larger study, the East London Prospective Cohort Study, previously published elsewhere [39], which focused on evaluating the maternal and infant outcomes of the prevention of mother-to-child transmission programme in the Eastern Cape, South Africa.

### Study setting

The data was collected in three large hospitals, namely, the Frere, Cecilia Makiwane and Bisho hospitals, serving a combined population of about two million people of the central region of Eastern Cape Province, South Africa. Frere hospital is an urban tertiary hospital receiving patients from across the entire central region of the Eastern Cape. In addition, Cecilia Makiwane hospital is a semi-urban regional hospital located in Mdantsane Township and provides both district and specialised care to patients from Buffalo City, Amathole and Chris Hani districts. Bisho hospital is a district hospital and receives patients from the neighbouring rural primary health care facilities.

### Study participants

All the participants enrolled in the electronic database of the East London Prospective Cohort Study (ELPCS) (*N* = 1709) were included in this study. Participants were included in the study if they received HIV diagnosis before or during the index pregnancy and attended the study sites for delivery between first of September 2015 to 31st of May 2016. All eligible participants took part in the study. The electronic data sheet of the ELPCS consisted of five different components; socio-demographic characteristics, HIV and ART history, Tuberculosis screening and IPT eligibility, pregnancy history and foetal outcomes and prophylaxis. This sub-study focused solely on the implementation of the IPT guideline within the cohort of the pregnant women enrolled in the database. Participants answered a few questions on whether they received a tuberculin skin test (Mantoux test) at the clinic before or during the index pregnancy and also, whether or not they had returned within 48–72 h to read the results. Participants’ medical records were also checked to confirm that the results of the Mantoux tests had been documented in the medical records. Treatment sheets in the patients’ medical records were extracted to confirm whether isoniazid had been prescribed in the preceding 2 years, prior to the study. Additional data on the place of antenatal care, the place and type of residence were extracted from the medical records. The drainage ante-natal clinics to each delivery facility were categorised according to their catchment areas. Places of residence and ante-natal care were categorised as rural, semi-urban or urban based on the South African Census [40].

### Ethical consideration

We obtained ethical approval from the Walter Sisulu University Ethics Committee (Reference: 098/2014). Permission was granted by the Eastern Cape Department of Health as well as the clinical governance of the respective hospitals. Participants received an information sheet (written in English and IsiXhosa) detailing the purpose and process of the study. Participants, subsequently, signed a written informed consent demonstrating their willingness to participate in the study. Few participants under the age of 18 years gave assent for their voluntary participation in the study while their legal guardian gave written informed consent for the study. Each participant was assigned a unique identifier (code) to prevent double entry. In addition, participants’ names and other linked identifiers were excluded from the database in compliance with the participants’ rights to privacy and confidentiality of medical information.

### Data analysis

Data were verified for accuracy by the field supervisor during the period of data collection; data were exported into the Statistical Package for Social Sciences version 24.0 (SPSS, Chicago, IL, USA). Simple, descriptive statistics (means and proportions) were used to describe the baseline characteristics of the participants. Rates of uptake of IPT and performance of the Mantoux test were presented in bar charts. The indicators of the performance of Mantoux test and IPT were evaluated in a univariate analysis. Adjusted and unadjusted logistic regression were used to examine inequality in uptake of uptake of IPT and performance of the Mantoux test.

## Results

The mean age of participants was 29.63 (±6.2) years (range 14–47 years). The majority of them were single (69.5%), had grade 7 to 12 level of education (86.5%) and were unemployed (74.7%). Almost half (46.3%) of the participants resided in semi-urban locations while 34.2% of them resided in rural areas (Table 1). The majority of the participants knew their HIV status at booking (80.1%), and of those who knew their status, 71.9% were already on the highly active antiretroviral therapy.

**Table 1:** Baseline characteristics of the participants

Variables (*N* = 1709)	Frequency (n)	Percentage (%)
Age (years)^a^		
≤19	60	3.5
20–24	331	19.5
25–29	459	27.0
30–34	452	26.6
35–39	303	17.8
40–47	96	5.6
Marital Status		
Married	312	18.3
Single	1187	69.5
Cohabiting	186	10.9
Divorced	24	1.4
Place of Residence		
Rural	585	34.2
Semi-urban	792	46.3
Urban	332	19.4
Educational level		
No formal education	5	0.3
Grade 1–6	115	6.7
Grade 7–12	1479	86.5
Tertiary	110	6.4
Employment status		
Unemployed	1277	74.7
Employed	432	25.3

^a^Missing data on age (*n* = 8)

### Clinical characteristics of patients

A majority of the patients were diagnosed with HIV before the index pregnancy and most of them were already on anti-retroviral therapy (Table 2). Likewise, most of the respondents booked for antenatal care during the second trimester (71.9%), and in WHO stage one (87.8%).

**Table 2:** Clinical characteristics of the participants

Variables (*N* = 1709)	Frequency	Percent
Trimester at booking		
First	210	12.3
Second	1229	71.9
Third	270	15.8
Status at first antenatal booking		
Positive	1382	80.9
Negative	94	5.5
Unknown	233	13.6
On HAART at Booking		
Yes	998	58.4
No	711	41.6
WHO Clinical Stage		
Stage I	1500	87.8
Stage II	189	11.1
Stage III	16	0.9
Stage IV	4	0.2
CD4 at booking		
500 and above	478	28.0
200–499	733	42.9
Unknown	279	16.3
Less than 200	219	12.8
Viral load at booking		
Undetectable (< 50)	636	37.2
Low viremia (50–999)	185	10.8
Unknown	654	38.3
High viremia (≥1000)	234	13.7

*SD* Standard deviation, *HAART* Highly active anti-retroviral therapy, *WHO* World Health Organization

### Performance of Mantoux test

Overall, the Mantoux test was performed in slightly less than half of the pregnant women in the study (47.3%; *n* = 774); with considerably fewer Mantoux tests being performed in the rural health facilities (10%). Health facilities in the urban and semi-urban areas performed more Mantoux tests; 54.2 and 50.5%, respectively (Table 3).

**Table 3:** Indicators of performance of the Mantoux test and IPT

Variables	Performed	Not Performed	*p*-value
	n (%)	n (%)	
Mantoux Test by study sites			
Frere hospital (*n* = 608)	363 (59.7)	245 (40.3)	<0.001
Cecilia Makiwane hospital (*n* = 711)	379 (53.3)	332 (46.7)	
Bisho hospital (*n* = 319)	32 (10.0)	287 (90.0)	
Mantoux Test by type of residence			
Rural (*n* = 564)	220 (39.0)	344 (61.0)	<0.001
Semi-urban (*n* = 755)	381 (50.5)	374 (49.5)	
Urban (n = 319)	173 (54.2)	146 (45.8)	
INH prophylaxis by study sites			
Frere hospital (*n* = 631)	511 (81.0)	120 (19.0)	<0.001
CMH (*n* = 706)	592 (83.8)	117 (16.2)	
Bisho (*n* = 315)	206 (65.4)	109 (34.6)	
INH Prophylaxis by type of residence			
Rural (*n* = 568)	424 (74.6)	144 (25.4)	0.004
Semi-urban (*n* = 760)	622 (81.8)	138 (18.8)	
Urban (*n* = 324)	263 (81.2)	61 (18.8)	

INH=Isoniazid; Missing data were excluded from the analysis (there were missing data for 71 participants on Mantoux test and 57 missing data on INH Prophylaxis)

The results of adjusted and unadjusted logistic regression analyses are presented in Table 4. In the unadjusted model, demographic characteristics (age, marital status, place of residence, employment status and level of education) as well as the HIV status at first antenatal booking were independently associated with uptake of Mantoux testing. Clinical characteristics (CD4 count, viral load and trimester at antenatal care booking) were not significantly associated with uptake of Mantoux testing. However, in the adjusted model after adjusting for confounding factor (study site), negative status at first antenatal booking, place of residence and educational status were significantly associated with uptake of Mantoux test. Study sites confounds with place of residence and was not included in the models. Individuals who had grade 1–6 level of education were 49% less likely to have received a Mantoux test compared to those who had a tertiary level of education. Also, individuals who resided in rural areas were 37% less likely to have received a Mantoux test compared to those who resided in urban areas. The relationship between employment status and uptake of Mantoux test was not statistically significant after controlling for marital status, age and HIV status at booking.

**Table 4:** Adjusted and unadjusted logistic regression analysis showing socio-economic inequality in uptake of Mantoux Test

Variables (*N* = 1638)	Never tested	Got tested	UOR	AOR
Marital status				
Single	652 (57.2)	488 (42.8)	0.58 (0.45–0.75)***	0.93 (0.38–2.28)
Cohabiting	70 (40.7)	102 (59.3)	1.13 (0.78–1.66)	0.58 (0.24–1.39)
Divorce/separated	9 (40.9)	13 (59.3)	1.12 (0.47–2.71)	0.97 (0.39–1.19)
Married	133 (43.8)	171 (56.3)	1	1
Education status				
No formal Education	2 (40.0)	3 (60.0)	1.25 (0.20–7.76)	1.19 (0.19–7.67)
Grade 1–6	73 (67.0)	36 (33.0)	0.41 (0.24–0.71)*	0.51 (0.29–0.91)*
Grade 7–12	740 (52.3)	676 (47.7)	0.76 (0.51–1.12)	0.83 (0.55–1.25)
Tertiary	49 (45.4)	59 (54.6)	1	1
Place of Residence				
Rural	344 (61.0)	220 (39.0)	0.54 (0.41–0.71)***	0.63 (0.47–0.84)*
Semi-urban	374 (49.5)	381 (50.5)	0.86 (0.66–1.12)	0.91 (0.69–1.19)
Urban	146 (45.8)	173 (54.2)	1	1
Employment status				
Employed	191 (46.0)	224 (54.0)	1.44 (1.15–1.79)*	1.24 (0.98–1.56)
Unemployed	673 (55.0)	550 (45.0)	1	1
Age				
≥25 years	640 (51.0)	616 (49.0)	1.33 (1.05–1.68)*	1.26 (0.99–1.62)
<25 years	218 (58.0)	158 (42.0)	1	1
HIV Status at booking				
Positive	722 (54.2)	610 (45.8)	0.86 (0.65–1.15)	0.85 (0.63–1.15)
Negative	32 (36.4)	56 (63.6)	1.78 (1.07–2.97)*	1.70 (1.01–2.88)*
Unknown	110 (50.5)	108 (49.5)	1	1
Trimester at booking				
First	83 (48.3)	89 (51.7)	1.15 (0.78–1.69)	1.13 (0.76–1.68)
Second	644 (53.6)	557 (46.4)	0.93 (0.71–1.21)	1.02 (0.77–1.34)
Third	137 (53.6)	128 (48.3)	1	1
CD4 at booking				
500 and above	263 (56.9)	199 (43.1)	0.75 (0.54–1.04)	0.77 (0.54–1.09)
200–499	372 (52.2)	340 (47.8)	0.91 (0.67–1.23)	0.93 (0.68–1.28)
Unknown	122 (49.0)	127 (51.0)	1.03 (0.72–1.49)	1.06 (0.71–1.56)
Less than 200	107 (49.8)	108 (50.2)	1	1
Viral load at booking				
Undetectable (<50)	333 (53.8)	286 (46.2)	1.01 (0.74–1.38)	0.95 (0.68–1.32)
Low viremia (50–999)	97 (53.3)	85 (46.7)	1.03 (0.70–1.53)	0.94 (0.62–1.42)
Unknown	315 (51.1)	302 (48.9)	1.13 (0.83–1.54)	1.02 (0.73–1.41)
High viremia (≥1000)	119 (54.1)	101 (45.9)	1	1

***p-value < 0.001; *p-value < 0.005; UOR- Unadjusted Odds Ratio; AOR-Adjusted Odds Ratio

### Uptake of isoniazid prevention therapy

The rate of uptake of IPT was 79% (*n* = 1309). However, rural health facilities lagged behind facilities in semi-urban and urban centres; 65.4% versus 83.9% versus 81%, respectively. Pregnant women living in rural communities were the least likely to commence INH prophylaxis in comparison to those living in semi-urban or urban centres; 74.6% versus 81.8% versus 81.2%, respectively.

The results of the adjusted and unadjusted logistic regression analyses are presented in Table 5. In the unadjusted model, the place of residence and employment status were independently associated with uptake of INH prophylaxis. However, after controlling for important covariates, the place of residence and employment were no longer significantly associated with INH prophylaxis. Study sites confounds with place of residence and was not included in the models.

**Table 5:** Adjusted and unadjusted binary logistic regression models showing relationship between sociodemographic factors, clinical characteristics and uptake of INH Prophylaxis

Variables (*N* = 1652)	Did not receive INH	Received INH	UOR	AOR
Marital status				
Single	247 (21.7)	893 (78.3)	0.88 (0.64–1.20)	0.71 (0.23–2.18)
Cohabiting	32 (17.8)	148 (82.2)	1.12 (0.70–1.80)	0.68 (0.23–2.04)
Divorce/separated	4 (16.7)	20 (83.3)	1.21 (0.40–3.67)	0.80 (0.25–2.53)
Married	60 (19.5)	248 (80.5)	1	1
Education status				
No formal Education	1 (20.0)	4 (80.0)	0.97 (0.10–9.09)	1.53 (0.15–15.56)
Grade 1–6	29 (26.9)	79 (73.1)	0.66 (0.35–1.25)	0.72 (0.37–1.39)
Grade 7–12	292 (20.4)	1139 (79.6)	0.94 (0.58–1.54)	1.01 (0.61–1.68)
Tertiary	21 (19.4)	87 (80.6)	1	1
Place of Residence				
Rural	144 (25.4)	424 (74.6)	0.68 (0.49–0.96)*	0.72 (0.51–1.02)
Semi-urban	138 (18.2)	622 (81.8)	1.05 0.75–1.46)	1.07 (0.76–1.50)
Urban	61 (18.8)	263 (81.2)	1	1
Employment status				
Employed	271 (22.0)	959 (78.0)	1.37 (1.03–1.83)*	1.29 (0.96–1.74)
Unemployed	72 (17.1)	350 (82.9)	1	1
Age				
≥25 years	80 (21.2)	297 (78.8)	1.03 (0.78–1.37)	0.94 (0.70–1.27)
<25 years	263 (20.7)	1006 (79.3)	1	1
HIV Status at booking				
Positive	277 (20.7)	1062 (79.3)	1.04 (0.74–1.47)	1.03 (0.72–1.48)
Negative	18 (20.5)	70 (79.5)	1.06 (0.57–1.94)	1.00 (0.54–1.85)
Unknown	48 (21.3)	177 (78.7)	1	1
Trimester at booking				
First	51 (28.2)	130 (71.8)	0.65 (0.42–1.01)	0.66 (0.42–1.03)
Second	238 (19.7)	968 (80.3)	1.04 (0.75–1.45)	1.07 (0.77–1.51)
Third	54 (20.4)	211 (79.6)	1	1
CD4 at booking				
500 and above	91 (19.4)	377 (80.6)	1.21 (0.82–1.79)	1.18 (0.78–1.78)
200–499	145 (20.2)	573 (79.8)	1.15 (0.80–1.66)	1.13 (0.78–1.66)
Unknown	58 (23.3)	191 (76.7)	0.96 (0.62–1.48)	1.00 (0.63–1.57)
Less than 200	49 (22.6)	168 (77.4)	1	1
Viral load at booking				
Undetectable (<50)	112 (17.8)	517 (82.2)	1.41 (0.97–2.03)	1.30 (0.88–1.92)
Low viremia (50–999)	47 (25.7)	136 (74.3)	0.88 (0.56–1.39)	0.80 (0.50–1.27)
Unknown	131 (21.3)	482 (78.6)	1.12 (0.78–1.61)	1.11 (0.76–1.63)
High viremia (≥1000)	53 (23.3)	174 (76.7)	1	1

***P-value <0.001; *P-value <0.005; UOR- Unadjusted Odds Ratio; AOR-Adjusted Odds Ratio

## Discussion

The present study sought to evaluate the uptake of isoniazid prevention therapy including the performance of the Mantoux test along rural-urban divide in the Eastern Cape, South Africa. Intensive symptom screening for active TB and performance of the Mantoux test in HIV-infected individuals without TB disease have been the core strategies implemented by the National Department of Health of South Africa [41] in order to mitigate the scourge of the TB epidemic. These strategies have the potential to further reduce the incidence of TB and TB-associated morbidity and mortality in people living with HIV. In view of the trends of TB-associated mortality in pregnant women living with HIV in South Africa [3], implementation of preventive measures has become imperative in the country, especially in the Eastern Cape. In the current study, the performance of the Mantoux test was low; only 47% of the eligible pregnant women had undergone the test. This is particularly concerning for pregnant women residing in the rural areas who accessed the rural health facilities. The performance of the Mantoux test was low in the rural health facilities where only 10% of the pregnant women living with HIV received the test.

Overall, the uptake of the Mantoux test is still below the acceptable level, however, the rates observed in the urban and semi-urban areas are encouraging, and thus, give optimism for improvement with better strategies. The wide disparity between the urban and rural dwellers should be a cause for concern for the TB programme managers and the Eastern Cape Health Department. As observed in this study, lower educational attainment and rural dwelling were associated with performance of Mantoux test. The result is not surprising in view of the fact that rural poor people tend to access the nearest health facilities to them. Although, variations in the performance of the Mantoux test could be expected across health facilities and geographic settings, the gap observed between rural and urban health facilities in this study brings to the fore the issues of inequality of access to quality healthcare services in the country. Rural health care facilities face numerous challenges with TB screening and diagnosis; inadequate human and financial resources [42] and long waiting periods due to overcrowding. The health care challenges of rural dwellers in the study area have been documented previously [43]. The rural dwellers are often very poor, thus, access to urban health care facilities are unattainable because of long distance of travel and prohibitive transport costs. In addition, regular stock out of the purified protein derivative (PPD) for the Mantoux test has been a major problem in the region, and rural health facilities are worst hit. Therefore, the findings of this study provide a dashboard for future references on the performance of the Mantoux test in the Eastern Cape. Also, the results of this study further corroborate the urgency for revitalisation and re-engineering of the primary health care in the region, with particular emphasis on prevention of diseases in the rural, poor communities.

Regardless of the result of the Mantoux test, a high uptake rate of IPT (79%) was found in this study. This reinforces the optimism towards achieving the South African 2050 target of reducing the incidence and mortality due to TB; from 9800 per million in 2010 to 1500 per million in 2050 and 2180 per million in 2010 to 200 per million in 2050, respectively [32]. This rate is higher than the 34 and 32% uptake rate reported in some districts in Ethiopia [29, 30] and in Zimbabwe (54%) [33]. However, a similar uptake rate was reported by Omesa et al. [34] in an urban health district of Ethiopia (77%). This shows an improvement in the uptake of IPT in this setting and this could help to further reduce the morbidity and mortality associated with TB among HIV-infected pregnant women. However, there is still room for scale up of IPT implementation for all individuals living with HIV in the entire region, irrespective of geographic settings. While this study has provided a snapshot of the current situation of IPT implementation, periodic evaluation is imperative to ensure that poor performing health facilities can improve their service delivery to the community.

Though, this study found no association between the INH uptake and the HIV indicators in the logistic regression models; WHO clinical stage, CD4 count at booking and viral load, the epidemiological and biological link between the two infections are well documented [1–5, 10]. Given the rapid progression of latent to active TB during pregnancy [5], and its significant morbidity and mortality risks in HIV-infected pregnant women [1–4], this important preventive strategy should be accessible to all women living with HIV. However, clinicians initiating IPT in pregnant women should carefully monitor their patients for liver function adverse events, and grade 3 and above infant adverse events, given the report by Gupta et al. [25], which raised concerns on the safety of INH in this population.

Similar to the performance of the Mantoux tests, the uptake of IPT was higher in semi-urban and urban areas compared to rural areas. Though, the rural – urban disparity in the uptake of IPT was not statistically significant in the adjusted logistic regression model, the core issues around inequality in the quality of healthcare services across the socioeconomic divide cannot be ignored. The disparity between the performance of the Mantoux test and IPT initiation suggests that patients were initiated on the latter without the Mantoux test. HIV-infected pregnant women were more likely to be initiated on IPT without Mantoux test in the rural areas than in the urban areas. While this practice was supported by the directives of the South African National Department of Health during the period of stock out of PPD (Mantoux test) in the country, it was advised, however, that patients who received IPT should have the Mantoux test done as soon as it became available. It is however, commendable that pregnant women needing IPT might still receive the service in the rural health facilities regardless of Mantoux test. Hence, there is a good platform to begin health system strengthening in the rural communities of Eastern Cape. The rural health facilities more often than not, lack the resources (manpower and infrastructure) to adequately monitor and track patients to return for the Mantoux test. This challenge requires the attention of TB programme managers. There is an urgent need to scale up interventions to increase IPT uptake and ensure total coverage, particularly in the rural areas where access to quality healthcare is limited.

### Strengths and limitations

The three large maternity centres with their wide drainage antenatal clinics representing various demographic and geographic settings within the region gave credence to the findings of the study. Although the study provides a snapshot of the current performance of the health facilities in the region, periodic monitoring of IPT and the Mantoux test can potentially provide information on the trends and add greater value, hence, is recommended for the attention of TB programme managers. Despite the fact that the study participants live in communities similar to the entire country, the findings may not be generalizable to the entire country.

## Conclusion

The high uptake rate of isoniazid prevention therapy in pregnant women living with HIV in the study setting was commendable. However, the rate of performance of Mantoux test was generally low and worse in the rural health facilities. This study further highlights the inequality in the healthcare service delivery between rural and urban communities in the region. HIV-infected women with low educational attainment and residing in rural communities were less likely to have Mantoux test performed. Concerted efforts are needed to address the rural-urban inequality gaps in the roll-out of IPT and Mantoux test. TB programme managers should monitor both the Mantoux test and IPT rollout periodically with a view of reducing TB incidence and mortality in the region.

## Data Availability

All data are available from the corresponding authors upon reasonable requests.

## Abbreviations

ART: antiretroviral therapy
ELPCS: East London Prospective Cohort Study
HIV: human immunodeficiency virus
INH: isoniazid
IPT: isoniazid prevention therapy
TB: tuberculosis
TST: tuberculin skin test
